# Constructing a socioeconomic status index for colorectal cancer screening evaluation

**DOI:** 10.1093/eurpub/ckac131.108

**Published:** 2022-10-25

**Authors:** A Molina-Barceló, M Vanaclocha-Espí, M Pinto-Carbó, J Martín-Pozuelo, P Romeo, R Peiró-Pérez, C Barona, F Ortiz, A Nolasco, S Castán-Cameo

**Affiliations:** 1 Cancer and Public Health, FISABIO, Valencia, Spain; 2 Public Health, Regional Ministry of Health, Valencia, Spain; 3 Public Health, CIBER-ESP, Madrid, Spain; 4 Health Insurance Service, Regional Ministry of Health, Valencia, Spain; 5 Statistics, University of Alicante, Alicante, Spain

## Abstract

**Objective:**

To construct an individual socioeconomic status index (ISESI) with information available in the Population Information System of the Region of Valencia, Spain, and use it to analyse inequalities in a colorectal cancer screening programme (CRCSP).

**Methods:**

Cross-sectional study. The study population was composed of men and women aged between 50 and 69 who were invited to participate in the most recently completed round of the Region of Valencia CRCSP in 2020, n = 1,150,684. A multiple correspondence analysis was performed to aggregate information in the Segmented, Integrated and Geographical Population Analysis Code from the Population Information System of the Region of Valencia into an ISESI. Data from the 2016 Region of Valencia Health Survey was used for validation. The relationship between CRCSP participation and the ISESI was analysed by logistic regression models.

**Results:**

The variables included in the index were nationality, employment status, disability, healthcare coverage, risk of vulnerability and family size. The most important categories for determining the highest socioeconomic status were being employed and not being at risk of social vulnerability, and being unemployed and at risk of social vulnerability for determining the lowest socioeconomic status. Index validation demonstrated internal and external coherence for measuring socioeconomic status. The relationship between CRCSP participation and the ISESI categorised by quartile (Q) showed that Q4 (the lowest socioeconomic status) was less likely to participate OR = 0.769 (0.757-0.782) than Q1 (the highest socioeconomic status), and the opposite was found for Q2 OR = 1.368 (1.347-1.390) and Q3 OR = 1.156 (1.137-1.175).

**Conclusions:**

An ISESI was constructed and validated using Population Information System data and made it possible to evaluate inequalities in colorectal cancer screening.

**Key messages:**

• An individual socioeconomic status index was constructed and validated using Regional Population Information System data.

• The Individual socioeconomic status index constructed allows to systematically evaluates inequalities in colorectal cancer screening.

